# Instruments and scales for assessment, screening, and psychoeducational diagnosis of children and adolescents with autism spectrum disorder: a scoping review

**DOI:** 10.1590/1984-0462/2026/44/2025083

**Published:** 2026-10-19

**Authors:** Kleonara Santos Oliveira, Denise Brandão de Oliveira e Britto, Graziela Nunes Alfenas Fernandes, Stela Maris Aguiar Lemos

**Affiliations:** a Universidade Federal de Minas Gerais, Belo Horizonte, MG, Brazil.

**Keywords:** Autism spectrum disorder, Autistic spectrum disorder, Assessment instruments, Assessment scales, Psychoeducational diagnosis, Transtorno do espectro do autismo, Transtorno do espectro autista, Instrumentos de avaliação, Escalas de avaliação, Diagnóstico psicoeducacional

## Abstract

**Objective:**

To investigate instruments and scales for psychoeducational assessment, screening, and diagnosis of children and adolescents with autism spectrum disorder.

**Data source:**

This review followed the methodological procedures recommended by the Joanna Briggs Institute and the Preferred Reporting Items for Systematic Reviews and Meta-Analyses - Extension for Scoping Reviews (PRISMA-ScR). Its research question was, “What are the main instruments and scales for psychoeducational assessment, screening, and diagnosis of children and adolescents with autism spectrum disorder in school?”.

**Data synthesis:**

The study included 16 articles that investigated instruments for psychoeducational assessment, screening, and diagnosis of children and adolescents with autism spectrum disorder. The instruments found were the Psychoeducational Profile - Revised (PEP-R), Psychoeducational Profile 3 (PEP-3), and the Wechsler Intelligence Scale for Children (WISC-IV). This indicates a scarcity of instruments and scales for the psychoeducational assessment, screening, and diagnosis of children and adolescents with autism spectrum disorder.

**Conclusions:**

The most used instruments to assess the psychoeducational profile of schoolchildren with autism spectrum disorder include the Revised Psychoeducational Profile (PEP-R) and the Psychoeducational Profile, 3^rd^ Edition (PEP-3), according to scientific literature. There is a scarcity of instruments and scales for the psychoeducational assessment, screening, and diagnosis of children and adolescents with autism spectrum disorder, suggesting the need for further research on the subject.

## RESUMO

**Objetivo:**

Investigar os instrumentos e escalas para avaliação, rastreio e diagnóstico psicoeducacional de crianças e adolescentes com transtorno do espectro autista.

**Fontes de dados:**

Foram seguidos os procedimentos metodológicos recomendados pelo Joanna Briggs Institute e a extensão para revisões de escopo do Preferred Reporting Items for Systematic Reviews and Meta-Analysis (PRISMA-ScR). A pergunta norteadora da revisão foi: quais os principais instrumentos e escalas para avaliação, rastreio e diagnóstico psicoeducacional de crianças e adolescentes com o transtorno do espectro autista no contexto escolar?

**Síntese dos dados:**

Foram selecionados 16 artigos neste estudo, que auxiliaram na investigação dos instrumentos para avaliação, rastreio e diagnóstico psicoeducacional de crianças e adolescentes com transtorno do espectro autista. Os instrumentos encontrados foram: Perfil Psicoeducacional Revisado (PEP-R), Perfil Psicoeducacional 3 (PEP-3) e a Escala Wechsler de Inteligência para Crianças (WISC-IV). Isso indica escassez de instrumentos e escala para a avaliação, rastreio e diagnóstico psicoeducacional de crianças e adolescentes com o transtorno do espectro autista.

**Conclusões:**

Entre os instrumentos mais utilizados para avaliar o perfil psicoeducacional de escolares com transtorno do espectro autista estão o Perfil Psicoeducacional Revisado (PEP-R) e o Perfil Psicoeducacional 3^a^ edição (PEP-3), segundo a literatura científica. Há escassez de instrumentos e escalas para avaliação, rastreio e diagnóstico psicoeducacional de crianças e adolescentes com transtorno do espectro autista, o que sugere a necessidade de mais pesquisas sobre o assunto.

## INTRODUCTION

Autism spectrum disorder (ASD) is a neurodevelopmental disorder with a neurobiological basis, characterized by persistent impairments in communication and social interaction, associated with restricted and repetitive patterns of behavior.^1^ Initial signs can be observed between 12 and 24 months of a child’s life, manifesting as significant developmental delays or regression of previously acquired skills.^2^ The etiology of ASD is multifactorial and, since it does not present biological markers, the diagnosis is predominantly clinical, based on systematic observation and information provided by caregivers. It is also recommended that standardized instruments be used to qualify the diagnosis.^3^ ^,^ ^4^ ^,^ ^5^

The assessment of a child with ASD is not limited to diagnostic conclusions, but is essential for defining their psychoeducational profile and guiding individualized therapeutic and educational interventions and strategies.^1^ ^,^ ^3^ The assessment of the student’s psychoeducational profile aims to understand their learning, cognition, motor skills, behavior, and social relationships at school and in the ecological environment, integrating educational and psychological knowledge to obtain a complete overview of child development and offer the necessary references to assist in the development of an inclusive educational curriculum.^3^

According to the literature, instruments such as the Psychoeducational Profile - Revised (PEP-R) and the Psychoeducational Profile - 3 (PEP-3) prioritize the measurement of development in multiple domains, including expressive and receptive communication, cognition, motor skills, social interaction, and characteristic motor and verbal behavior, identifying strengths and weaknesses to guide educational interventions.^6^ ^,^ ^7^ Historically, children with ASD have sometimes been treated as “untestable” due to low adherence to testing situations, bonding difficulties, and expressive and receptive communication limitations, which hinder the applicability of conventional instruments.^7^ Thus, the PEP-R and later the PEP-3 were conceived as benchmarks for understanding the repertoire of skills and needs of children with ASD, supporting the definition of the level of support and the development of an individualized educational and clinical plan, as well as the possibility of short, medium, and long-term follow-up.^7^

The timely and thorough identification of ASD is a structural basis for implementing interventions capable of enhancing development and improving the quality of life of children and adolescents. However, the psychoeducational assessment of this population requires duly validated instruments and scales, sensitive to the specificities of the disorder and able to reliably screen, support diagnosis, and monitor outcomes with adequate adaptation to different cultural and educational contexts. Nevertheless, the literature still has a limited collection of validated measures for psychoeducational screening, diagnosis, and characterization, especially in specific settings. This weakens the standardization and reliability of practices and limits the conduct of interventions rigorously anchored in scientific evidence.

This scoping review aimed to identify instruments and scales for screening, assessment, and psychoeducational diagnosis of children and adolescents with ASD at school and to provide an updated overview to support education and health professionals in choosing appropriate instruments, contributing to the development of contextualized and effective practices.

## METHOD

This scoping review followed the guidelines of the Joanna Briggs Institute (JBI) and presented its results according to the Preferred Reporting Items for Systematic Reviews and Meta-Analyses - Extension for Scoping Reviews (PRISMA-ScR).^8^ The study had the following phases: developing the research question; identifying relevant studies; selecting studies; investigating data; and grouping, summarizing, and presenting the results.^9^

The review used the Population, Concept, and Context (PCC) strategy^10^ to include publications that covered topics related to:

a) the population: people with ASD aged 5 to 12 years;
b) the concept: psychoeducational profile;
c) context: schools, thus answering the research question: “What are the main instruments and scales for psychoeducational assessment, screening, and diagnosis of children and adolescents with autism spectrum disorder in school?”

The review considered publications that met the PCC inclusion criteria, in any language, from the last 13 years. The search was limited to the last 13 years because this period coincides with the publication of the Diagnostic and Statistical Manual of Mental Disorders, Fifth Edition - DSM-5 (2013), which introduced relevant changes in ASD criteria and classification, influencing the characterization of samples and the comparability between studies, and ensuring greater diagnostic consistency and up-to-date findings.

Articles were searched on the electronic databases of Latin American and Caribbean Health Sciences Literature (LILACS); Medical Literature Analysis and Retrieval System Online (MEDLINE, USA), via the United States National Library of Medicine (PubMed); Cochrane, via the Brazilian Coordination for the Improvement of Higher Education Personnel (CAPES) Portal; Scopus, via CAPES Portal; Embase, via CAPES Portal; and Web of Science, via CAPES Portal, from April to June 2024. The search terms, selected from the Health Sciences Descriptors (DeCS) and Medical Subject Headings (MeSH), were autism spectrum disorder, psychoeducational diagnosis. The study also used free terms: synonyms, related terms, orthographic variations, abbreviations, acronyms, and keywords indispensable to the research: diagnóstico psicoeducacional, psychoeducational diagnosis, psychoeducational assessment, educational psychology, educational assessment. The consulted databases and their respective equations are described in Table 1.

**Table 1.** Databases and respective search strategy.

Database	Strategy
LILACS	(“Transtorno do Espectro Autista” OR “Autism Spectrum Disorder” OR “Trastorno del Espectro Autista” OR “Trouble du spectre autistique” OR “Transtorno Autístico” OR “Autistic Disorder” OR “Trastorno Autístico” OR “Trouble autistique” OR Autismo OR Autism) AND (“Avaliação Educacional” OR “Educational Measurement” OR “Evaluación Educacional” OR “Évaluation des acquis scolaires” OR “Psicologia Educacional” OR “Psychology, Educational” OR “Psicología Educacional” OR “Psychologie de l’éducation” OR “Diagnóstico Psicoeducacional” OR “Psychoeducational Diagnosis” OR “Psychoeducational Assessment” OR “Educational Psychology” OR “Educational Assessment”)
MEDLINE via PubMed	(“Autism Spectrum Disorder” OR “Autistic Disorder” OR Autism) AND (“Educational Measurement” OR “Psychology, Educational” OR “Psychoeducational Diagnosis” OR “Psychoeducational Assessment” OR “Educational Psychology” OR “Educational Assessment”)
COCHRANE (via Capes Portal)	(“Autism Spectrum Disorder” OR “Autistic Disorder” OR Autism) AND (“Educational Measurement” OR “Psychology, Educational” OR “Psychoeducational Diagnosis” OR “Psychoeducational Assessment” OR “Educational Psychology” OR “Educational Assessment”)
SCOPUS (via Capes Portal)	(“Autism Spectrum Disorder” OR “Autistic Disorder” OR Autism) AND (“Educational Measurement” OR “Psychology, Educational” OR “Psychoeducational Diagnosis” OR “Psychoeducational Assessment” OR “Educational Psychology” OR “Educational Assessment”)
Web of Science (via Portal Capes)	(“Autism Spectrum Disorder” OR “Autistic Disorder” OR Autism) AND (“Educational Measurement” OR “Psychology, Educational” OR “Psychoeducational Diagnosis” OR “Psychoeducational Assessment” OR “Educational Psychology” OR “Educational Assessment”)
EMBASE (via Capes Portal)	(autism) AND (‘psychoeducational assessment’ OR ‘educational psychology’ OR ‘educational assessment’)

Source: The authors.

MEDLINE data were excluded from the Virtual Health Library (VHL) of the Ministry of Health, as they were analyzed via PubMed. Duplicates were excluded as the search was finalized within each database.

The review selected articles from the last 13 years, encompassing children aged 5 to 12 years, and excluded those that did not answer the research question, as well as review articles, doctoral theses, master’s theses, essays, conference proceedings, case studies, and expert opinion articles (Figure 1).

**Figure 1.**
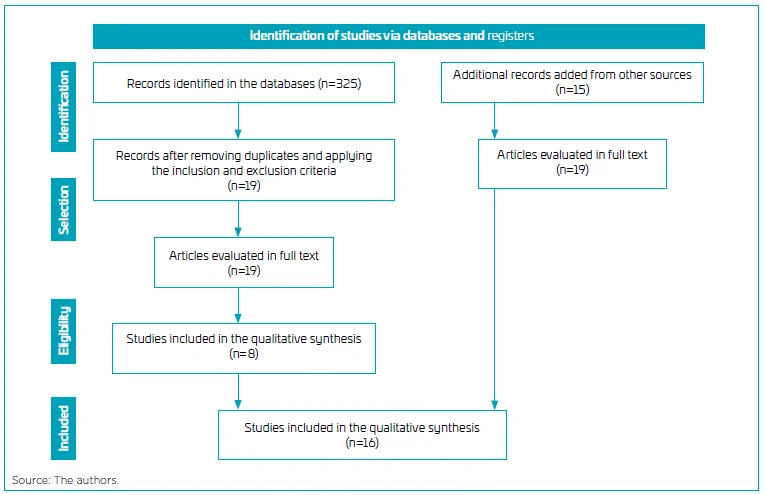
Flowchart of the article selection process.

Two researchers identified and screened the articles by reading the titles and abstracts independently. The reference generator (Rayyan QCRI^®^) was used to organize research results, remove duplicates, and help with selection. Then, the researchers compared their analyzed data; in case of disagreement or incompatibility, they reanalyzed them until they reached a common result. Next, an evaluator read the selected eligible articles in full and excluded those that did not meet the article selection criteria.

The following article data were extracted to an Excel^®^ spreadsheet: authors and year, design and country, sample (subjects, sex, age, and total sample size), diagnosis, instruments, and main results.

## RESULTS

The initial database search resulted in 325 articles. After excluding duplicates, reading titles and abstracts, and applying exclusion criteria, 19 articles remained for full-text reading. Then, 11 articles were excluded because they did not meet the inclusion criteria. Eight articles remained and were included in the review after qualitative synthesis. Subsequently, other articles on psychoeducational profile and autism were manually searched on Google Scholar with the descriptors: psychoeducational profile and autism spectrum disorder, considering articles from the last 13 years, in any language. Hence, another eight articles were selected, read in full, and included in the study, and a total of 16 articles were selected (Figure 1, Table 2).

**Table 2.** Summary of studies included in the review.

Reference	Design	Country	Participants	Sex	Age	Total N	Diagnosis	Instruments	Main results
Lopes-Herrera et al.^11^	Cross-sectional study	Brazil	Children	-----	2 to 9 years	15 verbal; 15 nonverbal	ASD	PEP-R	Verbal children performed better than nonverbal children.
Silva et al.^12^	Retrospective observational study	Brazil	Children	-----	3 years and 8 months to 10 years and 8 months	16	ASD	PEP-R	ABA-based therapy enabled autistic children to develop academic, social, and daily living skills.
Gomes et al.^13^	Cross-sectional study	Brazil	Children	-----	3 years and 2 months to 8 years and 10 months	24	ASD	PEP-R	It is feasible to use these technologies to empower caregivers and improve the development of children with autism.
Chen et al.^14^	Longitudinal cross-sectional study	China	Children	-----	3 to 7 years	107	ASD	PEP-3	Cognition, language expression, language comprehension, emotion, and social interaction in the developmental behavior subtest of the PEP-3.
Craig et al.^15^	Cross-sectional study	Italy	Children	-----	3 to 6 years	46	ASD	PEP-3	Children with ASD have greater motor impairments correlated with social communication skills.
Weismer et al.^16^	Case-control study	USA	Children	-----	4 to 8 years	22	ASD	WISC-IV	Children with ASD have significant deficits in all components of executive functions.
Yu et al.^17^	Case-control study	China	Children	466 boys 88 girls	2 years to 7 years and 9 months	554	ASD	PEP-3	The study strongly supports the reliability and validity of PEP-3, which can be used in mainland China.
Gomes et al.^18^	Cross-sectional study	Brazil	Children	-----	1 year and 11 months to 8 years and 10 months	10	ASD	PEP-R	The results indicated developmental gains for all children, although to varying degrees.
Pittala et al.^19^	Longitudinal cross-sectional study	France	Children	-----	2 to 6.6 years	20	ASD	PEP-R	After 2 years of intervention, the children improved significantly in PEP-R domains.
Halayem et al.^20^	Cross-sectional study	Tunisia North-Africa	Children	-----	3 to 7 years	50	ASD	PER-R	Pain sensitivity decreased in 40% and increased in 4% of children. Lower perception score on the PEP-R (p = 0.012).
Dubovyk^21^	Randomized clinical trial	Ukraine	Children	-----	4 to 6 years	79	Tics	PEP-R	A delay in the development of gross motor skills was noted in children with ASD and comorbid tic disorders compared to the control group, using the PEP-R.
Wang et al.^22^	Randomized clinical trial	China	Children	-----	4 to 6 years	70	ASD	PEP-3	At the 6-month follow-up, the PEP-3 scores of the treatment group were significantly higher than those of the control group.
Sun et al.^23^	Case-control study	China	Children	12 girls 100 boys	2 to 6 years	83: case 29: control	ASD	PEP-3	Cognitive verbal/preverbal language, receptiveness, affective expression, and communication improved significantly, as assessed through the PEP-3.
Shek et al.^24^	Longitudinal study	China	Children	-----	2 years to 7 years and 9 months	455	ASD	PEP-3	The internal consistency and retest reliability of the PEP-3 subtests were good to excellent.
Coelho et al.^25^	Longitudinal cross-sectional study	Portugal	Children	-----	3 to 6 years	30	ASD	PEP-R	Statistically significant differences between the groups with the best and worst outcomes in the assessment performed with PEP-R.
Paynter et al.^26^	Cross-sectional study	Australia	Children	-----	2 to 6 years	10	ASD	PEP-R	Children’s gross motor, cognitive, and verbal subscales improved significantly in the overall PEP-R score.

Note: ABA: applied behavior analysis; ASD: autism spectrum disorder; N: number; PEP-R: Psychoeducational Profile-Revised; PEP-3: Psychoeducational Profile, Third Edition; USA: United States of America; WISC-IV: Wechsler Intelligence Scale for Children-Fourth Edition. In the Sex column, “Not reported” indicates that sex was not specified in the source study; “Both sexes; numbers not reported” indicates inclusion of male and female participants without distribution by sex.

Source: The authors.

Four of the studies selected for this scoping review were published in Brazil, five in China, and one each in the United States, Australia, Italy, France, Tunisia, Ukraine, and Portugal. Nine of the 16 articles selected for analysis were cross-sectional observational studies, three were case-control studies, two were randomized clinical trials, one was a retrospective observational study, and one was a longitudinal study. The articles were published between 2013 and 2024.

China and Brazil had the highest number of publications on the psychoeducational assessment of children with ASD. The most frequently cited instruments for psychoeducational assessment were the PEP-R and PEP-3; one article cited the Wechsler Intelligence Scale for Children (WISC-IV). Thus, among the 16 selected articles, the identified assessment instruments were the PEP-R, PEP-3, and WISC-IV.

Research describes PEP-R as an instrument that measures the developmental age of children with ASD or related communication disorders.^7^ This instrument was organized into two scales: one that observes development and the other related to behavior, based on the CARS scale^7^ and Creak’s criteria.^7^ In Brazil, the PEP-R began to be used in 1992 for clinical purposes in its original, translated form.

PEP-3, which replaced PEP-R, assesses schoolchildren’s psychoeducational profile. It identifies irregular and idiosyncratic learning patterns through a developmental impairment profile.^7^ ^,^ ^8^ The test has ten subtests, six focused on developmental skills and four on maladaptive behaviors and is indicated for children aged two to 12 years. PEP-3 data provide information on developmental skill levels and diagnosis in children and adolescents and help determine the level of support for ASD (the latter identified in children up to seven years old).^7^ The skills investigated include verbal and preverbal cognition, expressive language, receptive language, fine and gross motor skills, visuomotor imitation, expression of affectivity, social reciprocity, motor and verbal characteristics, behavioral problems, personal care, and adaptive behavior. Research also used the WISC-IV, albeit less frequently. It is a clinical instrument for assessing intelligence and problem-solving processes in children aged six to 16 years and 11 months, applied individually and organized into four indices: verbal comprehension, perceptual organization, working memory, and processing speed.^11^ ^,^ ^12^

The PEP-R and PEP-3 can be applied by specialized professionals, including educators, occupational therapists, speech-language-hearing pathologists, and psychologists, provided they are trained in their use and interpretation. The WISC-IV, however, is meant to be used only by psychologists in clinical settings. Among the studies that cited PEP-R and/or PEP-3, five were published in China, four in Brazil, and one each in France, Tunisia, Ukraine, Portugal, and Australia.

Two bilingual Brazilian translators adapted the PEP-R for Brazil, changing some items for cultural adaptation. After translation, reliability values for developmental and behavioral dimensions ranged from 0.80 to 0.97.^7^ The articles that cited the PEP-R reported using it for assessing verbal and nonverbal behavior in children with ASD,^11^ testing academic and social skills and activities of daily living,^12^ and verifying child development in schoolchildren stimulated by caregivers in the family context.^13^

Studies also reported using it to monitor developmental progress over two years, with assessments at the beginning and end of the period, finding associations between improvement in test domains and in daily functioning.^19^ ^,^ ^27^ Other studies reported using it to assess motor development, speech, and cognition and to identify delays in general motor skills in individuals with ASD.^21^

Studies that used the PEP-3 were found mainly in China - five of the six articles that cited it were Chinese. These studies described PEP-3 application for psychoeducational profiling, treatment support, and the formulation of long-term rehabilitation plans.^14^ ^,^ ^17^ ^,^ ^28^ A study on the psychometric properties of a simplified Chinese version (sCPEP-3) was also described, reporting internal consistency, test-retest reliability, interrater reliability, convergent validity, construct validity, and factorial validity.^17^ ^,^ ^22^ ^,^ ^23^ Furthermore, it was mentioned that the PEP-3 includes a caregiver report, which is absent in the PEP-R.^16^

## DISCUSSION

The assessment of children and adolescents with ASD is a complex process that must be multidisciplinary, with clinical observation, interviews with primary caregivers, and standardized instruments, such as tests and scales. Because it is multidisciplinary, it requires a partnership between education and health professionals, allowing for a detailed analysis of the child.

There was a greater concentration of international studies on the subject, with China and Brazil standing out in terms of the volume of publications. This pattern suggests a geographical asymmetry in scientific production and may reflect differences in the availability of instruments, the tradition of using the TEACCH/PEP model, and investment in psychoeducational assessment.

The studies highlight the importance of a psychoeducational assessment to support intervention plans and reassess the condition, which reinforces the centrality of functional and developmental measures in monitoring schoolchildren with autism spectrum disorder, beyond diagnostic classifications.

According to the studies analyzed, professionals can outline individualized intervention plans based on psychoeducational assessment, integrating specific therapies and strategies aligned with the child’s needs in school and clinical contexts. Timely and personalized intervention, based on multidisciplinary assessment and considering the particularities of each student, can enhance the development of children and adolescents with ASD, promote functioning in their daily lives, optimize quality of life, and expand opportunities for social and educational inclusion.

The literature synthesized in this study highlights the relevance of the PEP-3, which succeeded the PEP-R, as a resource that is widely used in different countries for characterizing the psychoeducational profile of students with ASD. In addition to supporting the early identification of needs, the PEP-3 provides relevant information for the planning, monitoring, and reassessment of school and clinical interventions.

Although this review highlighted the relevance of the PEP-R and PEP-3 for the psychoeducational assessment of children and adolescents with ASD, some limitations deserve attention. Most of the studies analyzed were conducted in very specific contexts, which limits the extrapolation of the results. Moreover, issues related to the accessibility and practical applicability of these instruments, such as costs, the need for specialized training, and linguistic and cultural barriers, were not sufficiently addressed. The study also did not perform a comprehensive comparative analysis between the PEP-R/PEP-3 and other available assessment tools, which restricts understanding of their relative advantages and limits the choice of more appropriate instruments for different contexts. As it is a scoping review, the synthesis is predominantly descriptive. Furthermore, the studies had heterogeneous designs, samples, and outcomes, which makes direct comparisons difficult and prevents robust quantitative estimates.

Despite its limitations, this review provides significant advances by consolidating evidence on the importance and functioning of the PEP-R and PEP-3 in psychoeducational assessment of children and adolescents with ASD. Both instruments effectively assessed multiple skills, such as verbal and nonverbal communication, fine and gross motor skills, social reciprocity, preverbal behavior, and visuomotor imitation. Moreover, the PEP-3 stands out for assessing signs of ASD, estimating developmental age, and identifying the level of support required, enabling the planning of individualized interventions and longitudinal monitoring of clinical progress. The versatility and scope of the instruments highlight their value in clinical and educational practice in different international contexts.

Further studies are needed to expand the cross-cultural validation and adaptation of the PEP-R and PEP-3 in more diverse contexts, including populations with socioeconomic and cultural differences, where resource limitations may interfere with their applicability. Rigorous comparative research between these instruments and other assessment tools could provide a more robust basis for selecting more effective methods. Longitudinal studies are also recommended to explore their performance across different age ranges and developmental stages, providing insights into their usefulness in the medium and long term.

It is also worth highlighting the importance of initiatives that democratize access to the instruments, such as the development of more accessible versions and widely available training programs, which are crucial to ensuring that professionals in different contexts can use them effectively.

## CONCLUSIONS

In conclusion, the investigation of instruments and scales for psychoeducational assessment, screening, and diagnosis of children and adolescents with ASD revealed that the PEP-R and PEP-3 are the most used such instruments, whereas the WISC-IV was mentioned only once. The study showed a scarcity of studies presenting instruments for assessing the psychoeducational profile of children and adolescents with ASD, suggesting the need for more research on the subject.

## Data Availability

The database that originated the article is available with the corresponding author.

## References

[1] Silva CC, Elias LC (2020). Intrumentos de avaliação no transtorno do espectro autista: uma revisão sistemática. Aval Psicol.

[2] American Psychological Association (2014). Manual diagnóstico de transtornos mentais: DSM-V.

[3] Zanon RB, Backes B, Bosa CA (2017). Autism diagnosis: relation among contextual, family and child factors. Psicol Teor Prat.

[4] Machado FP, Lerner R, Novaes BC, Palladino RR, Cunha MC (2014). Questionário de indicadores clínicos de risco para o desenvolvimento infantile: avaliação da sensibilidade para transtornos do espectro do autismo. Audiol Commun Res.

[5] Fernandes CS, Tomazelli J, Girianelli VR (2020). Diagnóstico de autism no século XXI: evolução dos domínios nas categorizações nosológicas. Psicol USP.

[6] Maia KS, Assumpção FB (2021). Escala de rastreio para transtorno no espectro autista: um estudo de validade para adolescentes e adultos. Bol Acad Paul Psicol.

[7] Leon V, Bosa C, Hugo C, Hutz CS (2004). Propriedades psicométricas do perfil psicoeducacional revisado: PEP-R. Aval Psicol.

[8] Cordeiro L, Soares CB (2020). Revisão de escopo: potencialidades para a sítese de metodologias utilizadas em pesquisa primária qualitativa. BIS.

[9] Peters M, Godfrey C, McInerney P, Soares CB, Khalil H, Parker D, Aromataris E, Munn Z (2024). JBI Reviewer’s Manual.

[10] Peters MD, Godfrey C, McInerney P, Munn Z, Tricco AC, Khalil H, Aromataris E, Munn Z (2020). JBI Reviewer’s Manual.

[11] Lopes-Herrera SA, Costa DG, Santos TR, Martins A (2023). Comparison between the socio-educational profiles of children with verbal and non-verbal Autism Spectrum Disorder. Codas.

[12] Silva AP, Bezerra IM, Antunes TP, Cavalcanti MP, Abreu LC (2023). Applied behavioral analysis for the skill performance of children with autism spectrum disorder. Front Psychiatry.

[13] Gomes CG, Silveira AD, Estrela LP, Figueiredo AL, Oliveira AQ, Oliveira IM (2021). Effects of the use of information and communication technologies on the training of caregivers of children with autism. Rev Bras Educ Espec.

[14] Chen GZ, Chang WL, Tan WW, Lin YY, Huang LL, Chen M (2022). Research on PEP-3 psychological education evaluation system for disabled children and autistic children in minority areas. Eur Rev Med Pharmacol Sci.

[15] Craig F, Crippa A, Ruggiero M, Rizzato V, Russo L, Fanizza I (2021). Characterization of Autism Spectrum Disorder (ASD) subtypes based on the relationship between motor skills and social communication abilities. Hum Mov Sci.

[16] Weismer SE, Kaushanskaya M, Larson C, Mathée J, Bolt D (2018). Executive function skills in school-age children with autism spectrum disorder: association with language abilities. J Speech Lang Hear Res.

[17] Yu L, Zhu X, Shek DT, Zou XB, Deng HZ, Au Yeung PW (2019). Validation of the simplified Chinese psychoeducational profile third edition in Mainland China. J Autism Dev Disord.

[18] Gomes CGS, Souza DG, Silveira AD, Oliveira IM (2017). Intervenção comportamental precoce e intensiva com crianças com autismo por meio da capacitação de cuidadores. Rev Bras Educ Espec.

[19] Pittala ET, Saint-Georges-Chaumet Y, Favrot C, Tanet A, Cohen D, Saint-Georges C (2018). Clinical outcomes of interactive, intensive and individual (3i) play therapy for children with ASD: a two-year follow-up study. BMC Pediatr.

[20] Halayem S, Charfi N, Touati M, Mrabet A, Bouden A (2018). Sensitivity to pain in autistic spectrum disorders: its links with self-gressivity. Tunis Med.

[21] Dubovyk K (2018). Prevalence of motor disorders and repetitive movements among children with autism spectrum disorders and Tics disorders. PMGP.

[22] Wang M, Jiang L, Tang X (2017). Levetiracetam is associated with decrease in subclinical epileptiform discharges and improved cognitive functions in pediatric patients with autism spectrum disorder. Neuropsychiatr Dis Treat.

[23] Sun C, Zou M, Zhao D, Xia W, Wu L (2016). Efficacy of folic acid supplementation in autistic children participating in structured teaching: an open-label trial. Nutrients.

[24] Shek DT, Yu L (2014). Construct validity of the Chinese version of the Psycho-Educational Profile-3rd Edition (CPEP-3). J Autism Dev Disord.

[25] Coelho AM, Aguiar A, Nunes C (2013). Evolução das perturbações do espectro do autismo. Possíveis factores preditivos?. Acta Pediatr Port.

[26] Paynter J, Scott J, Beamish W, Duhig M, Heussler H (2012). A pilot study of the effects of an Australian centre-based early intervention program for children with autism. Open Pediatr Med J.

[27] Souza JF, Gomes CG, Silveira AD, Rocha BA, Oliveira IM (2018). Efeitos de dois anos de intervenção comportamental intensiva no desenvolvimento de crianças com autismo. Abstracts of the 8^th^ Congresso Brasileiro de Educação Especial.

[28] Giacomo A, Craig F, Cristella A, Terenzio V, Buttiglione M, Margari L (2016). Can PEP-3 provide a cognitive profile in children with ASD? A comparison between the developmental ages of PEP-3 and IQ of Leiter-R. J Appl Res Intellect Disabil.

